# Solid-phase enzyme catalysis of DNA end repair and 3′ A-tailing reduces GC-bias in next-generation sequencing of human genomic DNA

**DOI:** 10.1038/s41598-018-34079-2

**Published:** 2018-10-26

**Authors:** Aihua Zhang, Shaohua Li, Lynne Apone, Xiaoli Sun, Lixin Chen, Laurence M. Ettwiller, Bradley W. Langhorst, Christopher J. Noren, Ming-Qun Xu

**Affiliations:** New England Biolabs, Inc., 240 County Road, Ipswich, MA 01938 USA

## Abstract

The use of next-generation sequencing (NGS) has been instrumental in advancing biological research and clinical diagnostics. To fully utilize the power of NGS, complete, uniform coverage of the entire genome is required. In this study, we identified the primary sources of bias observed in sequence coverage across AT-rich regions of the human genome with existing amplification-free DNA library preparation methods. We have found evidence that a major source of bias is the inefficient processing of AT-rich DNA in end repair and 3′ A-tailing, causing under-representation of extremely AT-rich regions. We have employed immobilized DNA modifying enzymes to catalyze end repair and 3′ A-tailing reactions, to notably reduce the GC bias observed with existing library construction methods.

## Introduction

Next-generation sequencing (NGS) has revolutionized both biology and medical diagnosis^1,2^. The large-scale parallel sequencing techniques permit genome wide analysis of disease development, prognosis, and drug response^3–5^. NGS analysis relies on preparation of a representative, non-biased library (a pool of DNA or RNA molecules) evenly distributed across the entire genome (or region). However, biases found in current methods of NGS library preparation can produce uneven coverage, compromising the quality of NGS analysis^6,7^. For example, some genomic sequences are over-represented whereas other regions have little or no coverage. Investigation of the technical and methodological sources of this GC content associated bias is critical to developing solutions to improve library quality and data analysis^8^.

Various sample treatment steps, including library construction, amplification and the sequencing chemistry itself can introduce GC bias^9^. It is widely accepted that library amplification via polymerase chain reaction (PCR) introduces bias in sequencing coverage due to uneven amplification of sequences with different GC content by DNA polymerases^6,10^. Interestingly, depletion of the high GC content regions, but not the high AT content regions, can largely be prevented by optimization of PCR conditions^6^. In addition, under-representation of AT-rich regions can only be slightly improved by avoiding library amplification^6,7^. Thus, bias against the AT-rich regions could be introduced prior to the amplification step or by sequencing chemistry. It is of interest to investigate whether a systematic bias against AT-rich sequences is introduced during library preparation.

A typical protocol of amplification-free library preparation for the Illumina platform comprises fragmentation, end repair (blunting and 5′ phosphorylation), 3′ A-tailing and adaptor ligation^11^. For streamlined protocols, once the sample DNA has been randomly sheared, the fragment ends are repaired by blunting and 5′ phosphorylation with a mixture of enzymes, such as T4 polynucleotide kinase (PNK) and T4 DNA polymerase (T4 DNA pol). This end repair step is followed by 3′ A-tailing at 37 °C using a mesophilic polymerase such as Klenow Fragment 3′-5′ exonuclease minus^11^, or at elevated temperatures using a thermophilic polymerase such as Taq DNA polymerase (Taq DNA pol)^12,13^. The identification of the key contributory factors responsible for generating sequence bias in these enzymatic steps is challenging, as multiple DNA modifying enzymes and a broad range of substrates are involved.

**Figure 1:**
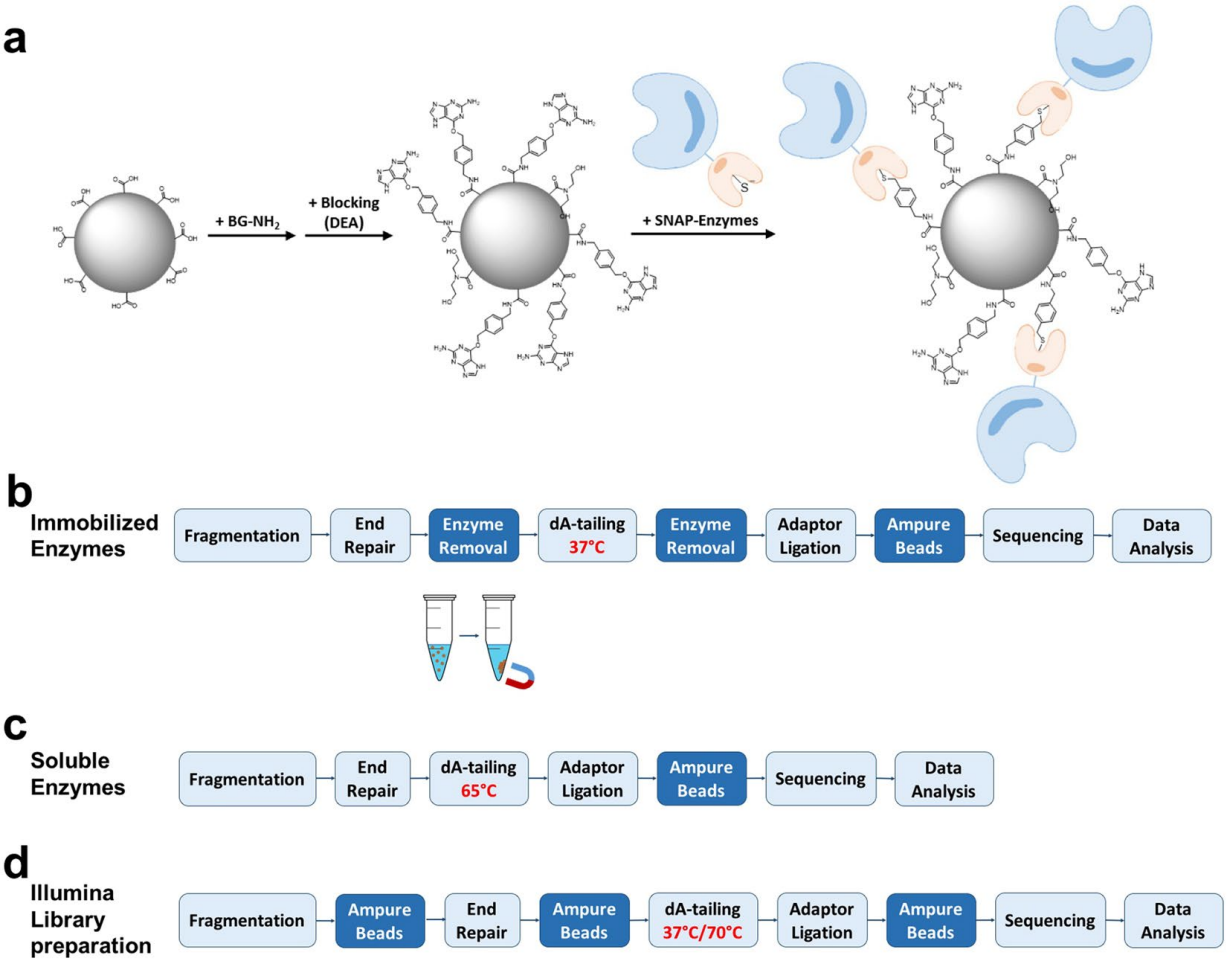
Enzyme immobilization and comparison of Illumina library preparation protocols. (**a**) A schematic of covalent conjugation of SNAP-tagged enzyme fusion proteins to magnetic beads functionalized with O^6^-benzylguanine (BG) moieties that specifically react with active site cysteine residues of SNAP-tag proteins, forming a stable covalent thioether bond^15,16^. (**b**) Workflow for library construction using immobilized enzymes for Illumina sequencing. A typical streamlined protocol for Illumina library construction is modified by employing immobilized enzymes to catalyze end repair and 3′ A-tailing. This method utilizes SNAP-tagged T4 DNA pol and PNK covalently conjugated to BG-functionalized magnetic beads to carry out end repair of fragmented DNA at 20°C (or 37 °C) for 30 min. The enzymes are removed by magnetic separation from the DNA pool, which is subjected to 3′ A-tailing at 37 °C for 30 min using immobilized Taq DNA pol. (**c**) Streamlined protocol for Illumina amplification-free library preparation using soluble enzymes. Typically, end repair and 3′ A-tailing of fragmented DNA are catalyzed by an enzyme mixture at 20 °C for 30 min, followed by heat treatment at 65 °C for 30 min. (**d**) The workflow of Illumina TruSeq DNA PCR-free LT Library Prep Kit with a purification step. End repair is performed for 30 min at 30 °C, followed by a bead-based step for clean up and size selection. 3′ A-tailing is carried out for 30 min at 37 °C with a subsequent treatment for 5 min at 70 °C. Each library was ligated to preannealed full-length paired-end Illumina adaptors, size-selected and analyzed, and sequenced on an Illumina sequencing platform.

In this report, we assessed the enzymatic reactions utilized in construction of amplification-free human DNA libraries for the Illumina sequencing platform (Supplementary Fig. 1). We first reproduced the GC-bias in enzymatic end-polishing (end repair and 3′ A-tailing) using synthetic DNA substrates of varying GC-content. Our investigation indicated that end repair and incubation at elevated temperature during 3′ A-tailing can result in preferential depletion of AT-rich regions. We hypothesized that due to DNA thermal breathing AT-rich DNA fragments are subjected to nuclease-mediated degradation and other events leading to under-representation. To prove this hypothesis, we devised an enzyme immobilization strategy to remove enzymes and to circumvent the requirement for high temperature incubation by physically removing the enzymes (Fig. 1). Enzymes covalently conjugated to magnetic beads successfully catalyzed DNA end-polishing steps in library preparation for the Illumina sequencing platform at low temperature, leading to significantly increased coverage of high AT-content regions of human DNA libraries.

## Results

### Sequence bias generated by the end repair step

**Figure 2:**
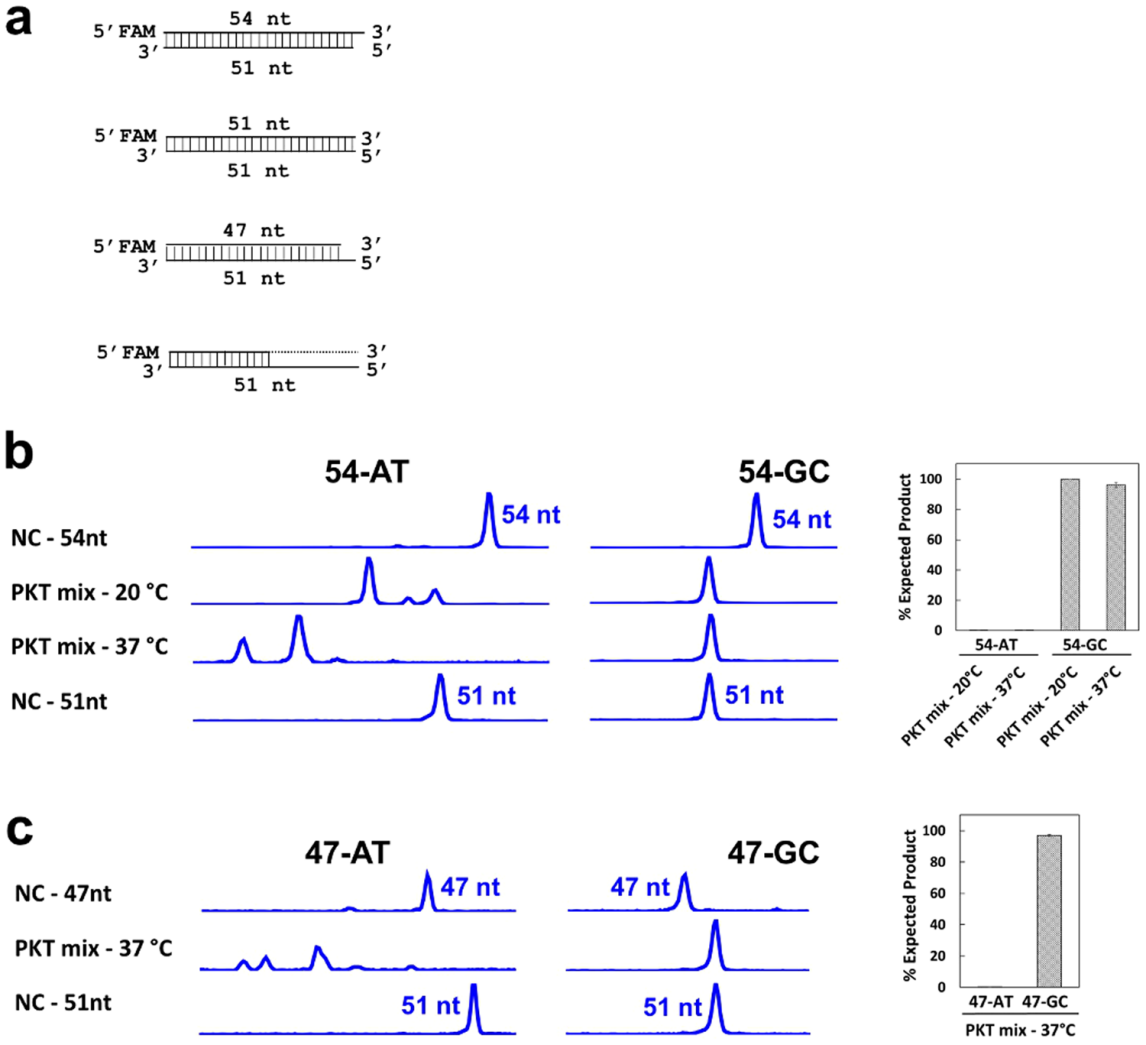
Exonuclease-mediated degradation of synthetic double-stranded AT-rich DNA by end repair enzyme mixture PKT using capillary gel electrophoresis (CE). (**a**) Illustration of various forms of synthetic substrates for study of DNA blunting, 5′ phosphorylation and 3′ A-tailing activities. Each double-stranded DNA substrate (dsDNA) comprises an oligonucleotide (oligo) possessing a 5′ FAM and a stretch of 3′ AT- or GC-rich terminal sequence, and a complementary oligomer as summarized in Supplementary Table 1. Blunt-end DNA can be generated either by polymerase activity to fill in a 3′ recessed substrate or 3′-5′ exonuclease activity to remove a 3′ overhang. Polymerization and nuclease activities can be detected using fluorescent synthetic DNA oligos by CE method^32^. 3′-5′ exonuclease activity of a DNA polymerase can generate a pool of shortened FAM-labeled oligos from dsDNA substrate, presumably due to high temperature that causes DNA end breathing. (**b**) Degradation analysis of enzyme mixture PKT using a pair of 5′ FAM-labeled synthetic DNA substrates containing a 3′ overhang. The CE data were analyzed by Peak Scanner software and the representative CE data are shown. PKT treatment of 54-AT at 20 °C and 37 °C resulted in detection of degradation species that are smaller than the 51 nt species (the expected blunting product). By contrast, the 54-GC reactions yielded a distinct 51 nt species under the same conditions. The negative control (NC) reactions (in the absence of enzyme) showed the positions of 54 nt and 51 nt strands. (**c**) Degradation study using a pair of 3′ recessed synthetic DNA substrates labeled with 5′ FAM. PKT treatment of 47-AT at 37 °C resulted in detection of degradation species that are smaller than the expected 51 nt product from primer extension whereas the negative control (NC) reactions showed the positions of 47 nt and 51 nt strands. Treatment of 47-GC, however, yielded the expected 51-nt product of polymerase activity with no detectable degradation of the FAM-labeled 47-nt strand.

The first step in our strategy was to examine end repair of synthetic model DNA substrates with either multiple terminal A-T base pairs or G-C base pairs (Supplementary Table 1 and Fig. 2a). We formulated an end repair mixture (“PKT”) comprising T4 DNA Pol, T4 PNK, and Taq DNA pol. DNA end repair reactions were carried out in the presence of dNTPs for 30 min at temperatures ranging from 20 °C to 37 °C. Figure 2 shows that treatment with the PKT mixture resulted in significant exonuclease degradation of the substrates possessing multiple terminal A-T base pairs (54-AT and 47-AT). In contrast, the substrates containing multiple terminal G-C base pairs (54-GC and 47-GC) were resistant to this exonuclease activity. Thus, the PKT mixture failed to properly blunt either 3′ recessed or 3′ protruding DNA substrates possessing AT-rich sequences. In addition, PKT treatment of three blunt-end substrates with various percentages of AT-content and the same 3′ terminal AT-rich sequence yielded degradation in a temperature-dependent manner (Supplementary Fig. 2).

We hypothesized that DNA degradation is caused by 3′-5′ exonuclease activity of T4 DNA pol. A synthetic blunt-end DNA substrate possessing a stretch of GC-rich sequence preceding nine terminal A-T base pairs was designed to aid in detection of exonucleolytic degradation in the presence of the PKT mixture or T4 DNA pol. Supplementary Fig. 3 shows that degradation was observed when the substrate was treated at 37 °C with T4 DNA pol alone, or with the PKT mixture, but no degradation occurred in the presence of only PNK, Taq DNA pol or PNK and Taq DNA pol together under the same conditions. Interestingly, the enzyme mixes displayed higher exonuclease activity when compared to that of T4 DNA pol alone, suggesting the other component(s) in the mixture can influence exonucleolytic activity. Indeed, the supplementation of either PNK or Taq DNA pol resulted in increased 3′-5′ exonuclease activity of T4 DNA pol. The data thus supports that 3′-5′ exonuclease activity of T4 DNA pol present in the enzyme mixes is capable of selective degradation of AT-rich DNA fragments during the progress of high temperature treatment.

### Sequence bias from 3′ A-tailing

We investigated if the terminal deoxynucleotidyl transferase activity of Taq DNA pol induces bias at the 3′ A-tailing step (Supplementary Fig. 4). Kinetic studies were performed using a pair of synthetic blunt-end substrates, possessing multiple terminal G-C (51-GC) or A-T (51-AT) base pairs, respectively. The kinetic rate of 3′ A-tailing at 65 °C is significantly higher for 51-GC than that for 51-AT (Supplementary Fig. 4a). In addition, a slight bias against the AT-rich end substrate was observed when the assays were performed at either 37 °C or 65 °C (Supplementary Fig. 4b–d). Insignificant 3′ A-tailing bias, however, was detected in the presence of higher amount of Taq DNA pol at 65 °C. It is plausible that the terminal transferase activity of Taq DNA pol displays preference for a 3′ terminal nucleotide (e.g. C over A). Alternatively, Taq DNA pol may favor a GC-rich terminal sequence for 3′ A-tailing due to formation of base-paired DNA terminal structure. We introduced mismatched terminal base pair(s) in 51-GC, by alteration of the 5′ terminal nucleotide(s) of the complementary sequence, and compared 3′ A-tailing efficiencies of the blunt-end, single mismatch or two base mismatch substrates. Supplementary Fig. 4e shows that 3′ A-tailing activity was completely blocked in the presence of either single or double base mispairing, indicating that Taq DNA pol cannot catalyze 3′ A-tailing of an unpaired terminal sequence.

Next, we investigated if polymerization and 5′-3′ flap endonuclease (5′ nuclease) activities of Taq DNA pol can play roles in depletion of AT-rich DNA at elevated temperatures. We examined a synthetic AT-rich DNA substrate by annealing two complementary oligos labeled with 5′ FAM and 3′ ROX, respectively, to monitor the fate of each oligo (Supplementary Fig. 5). Treatment of the AT-rich DNA substrate with Taq DNA pol or exonuclease-deficient Klenow Fragment at 65 °C, but not at 37 °C, resulted in detection of a product corresponding to primer extension activity of a DNA polymerase on the 5′ FAM-labeled strand. In addition, we also noticed the appearance of a product of approximately 28 nt corresponding to cleavage of the 3′ ROX-labeled strand present only in the sample treated at 65 °C, but not at 37 °C, with Taq DNA pol.

We further assessed whether Taq DNA pol can act on substrates with unpaired terminal sequences. Two DNA substrates were created by annealing oligos with nine unpaired bases at their termini (Supplementary Fig. 6). These substrates mimic terminal structures of DNA fragments when DNA end breathing occurs. The substrates were treated by Taq DNA pol at 37 °C or 65 °C. In each case, we detected a significant cleavage activity on the 3′ ROX-labeled strand corresponding to 5′ nuclease activity on the terminal unpaired substrates. Interestingly, the substrate possessing single-stranded GC-rich 3′ sequence yielded substantially more cleavage product at 37 °C and 65 °C compared to the substrate possessing single-stranded AT-rich 3′ sequence. Higher 5′ nuclease activity may be due to more efficient annealing of the GC-rich 3′ terminal sequence compared to the AT-rich 3′ sequence. Thus, we reason that when substantial end breathing of high AT-content fragments occurs at elevated temperatures during 3′ A-tailing reaction polymerization and 5′ nuclease activities of Taq DNA pol can result in depletion of the AT-rich DNA fraction in a DNA library, leading to sequence coverage bias.

### DNA end repair by immobilized enzymes

To circumvent high temperature treatment during library construction, we exploited an enzyme immobilization strategy based on SNAP-tag mediated covalent conjugation onto benzylguanine-functionalized magnetic beads (Fig. 1)^14–16^. The three DNA end polishing enzymes present in the PKT mix, T4 DNA Pol, PNK and Taq DNA Pol, were expressed as SNAP-tagged fusion proteins and purified. The SNAP-tagged enzymes were conjugated to magnetic beads and can be removed by magnetic separation from the reaction mixture. SNAP-tagged enzymes and their immobilized form were characterized for relevant end repair functions. Both soluble and immobilized T4 DNA pol displayed blunting activity on synthetic DNA substrates possessing either AT-rich and GC-rich terminal sequences (Supplementary Fig. 7a). Immobilized PNK exhibited 5′ phosphorylation activity although the specific activity is less than the soluble SNAP-tagged PNK (Supplementary Fig. 7b).

Kinetic study shows that immobilization of these SNAP-tagged enzymes resulted in lower enzymatic activity in comparison to their soluble counterpart, and required optimization of immobilization or reaction conditions to achieve efficient activity^17^. In addition, it was observed that 3′ A-tailing was not efficient in the buffer recommended for soluble Taq DNA pol. However, we found that immobilized Taq DNA pol displayed significantly higher 3′ A-tailing activity in the presence of polyethylene glycol (PEG), enabling efficient 3′ A addition at a moderate temperature, such as 37 °C (Supplementary Fig. 8).

**Figure 3:**
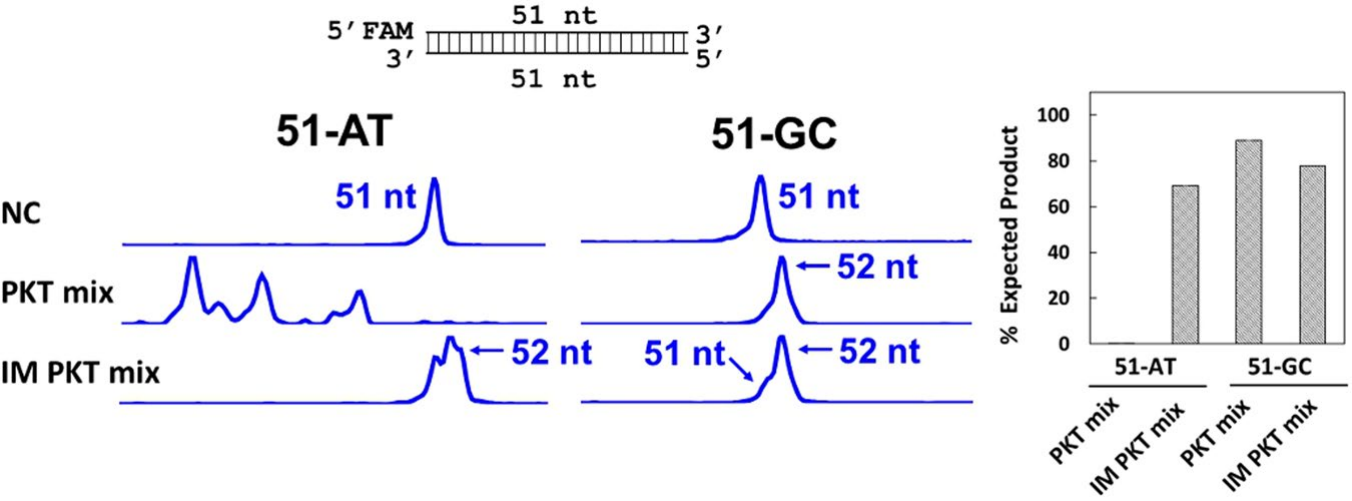
CE analysis of processing synthetic DNA by soluble enzyme mix PKT and immobilized enzymes. 5′ FAM-labeled blunt-end substrates, 51-AT possessing multiple 3′ terminal A-T base pairs, and 51-GC possessing multiple 3′ terminal G-C base pairs, were incubated with PKT for end repair at 20 °C for 30 min followed by 65 °C for 30 min (PKT mix). The substrates were also treated with immobilized T4 DNA pol and PNK at 20 °C for 30 min, followed by separation of the enzymes on beads and the reaction medium (supernatant). The reaction medium was subsequently treated with immobilized Taq DNA pol for 3′ A-tailing at 37 °C for 30 min (IM PKT mix). The CE data show that incubation with PKT resulted in extensive degradation of 51-AT and little degradation of 51-GC. Treatment of 51-AT or 51-GC with the immobilized enzymes resulted in mostly 3′ A-tailing product, without detectable degradation of the 5′ FAM-labeled oligos. NC, negative control reaction performed in the absence of enzyme.

To determine whether the degradation of AT rich DNA could occur during the end repair step, or if it was a consequence of high temperature treatment, we devised a workflow for Illumina library construction utilizing immobilized enzymes in place of their soluble counterparts for end repair and 3′ A-tailing (Fig. 1). This new approach involves incubation of the substrate with immobilized T4 DNA pol and PNK for 30 min at 20 °C. Enzymes are then removed by magnetic separation and 3′ A-tailing was performed by the addition of immobilized Taq DNA pol followed by incubation at 37 °C for 30 min. We first compared this new approach to a streamlined protocol which utilizes a high temperature incubation step for end repair and 3′ A-tailing (Fig. 3). Incubation with the soluble PKT mixture at 20 °C for 30 min and subsequent heat treatment at 65 °C for 30 min resulted in degradation of the AT-rich substrate. In contrast, the protocol utilizing immobilized enzymes resulted in predominantly 3′ A-tailed product from substrates possessing either AT-rich or GC-rich 3′ ends, without detectable degradation. The data indicates that a solid-phase catalysis approach is effective in end-polishing DNA fragments and avoids degradation of the AT-rich DNA.

### Reduced GC-bias in NGS libraries using immobilized enzyme protocol

**Figure 4:**
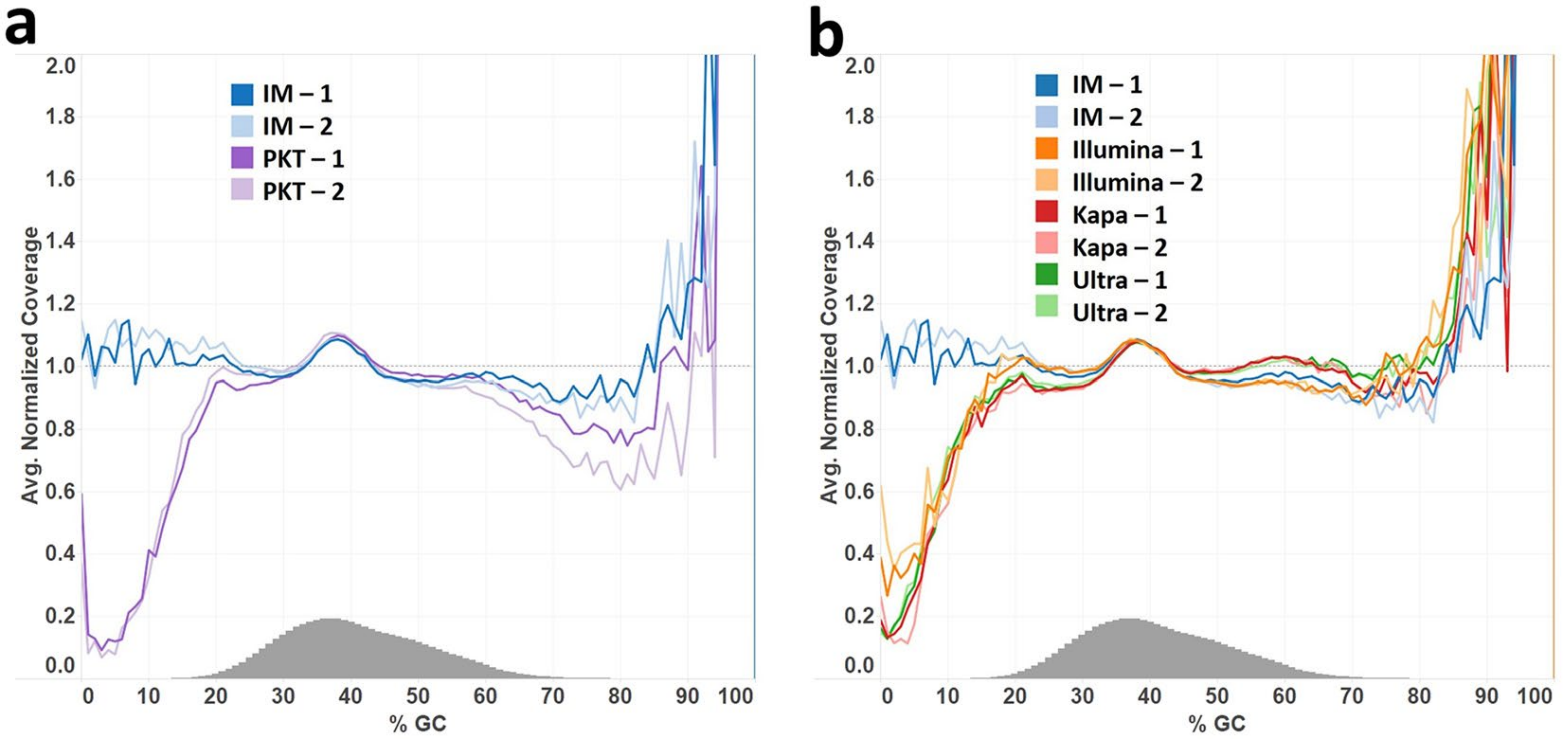
Genome-wide base composition bias curves in Illumina reads from PCR-free human DNA libraries. (**a**) The GC-bias curves from libraries (in duplicate) produced by the immobilized enzyme method (IM-1 and IM-2 in blue), for end repair for 30 min at 20 °C and 3′ A-tailing at 37 °C in contrast to the data from the libraries generated by the soluble enzyme method, with 3′ A-tailing at 65 °C, using enzyme mixture PKT (PKT-1 and PKT-2 in purple). (**b**) The GC-bias data of the immobilized enzyme method compared to the data from the duplicate libraries generated by Illumina TruSeq DNA PCR-free LT Library Preparation Kit (Illumina), Kapa Hyper Prep Kit (Kapa) or NEBNext Ultra II DNA Library Prep Kit for Illumina (Ultra) according to the protocols of the manufacturers. The Illumina protocol carries out end repair for 30 min at 30 °C and 3′ A-tailing for 30 min at 37 °C, followed by incubation at 70 °C for 5 min, and includes a clean-up and size selection step between end repair and 3′ A-tailing. The Kapa Hyper and NEBNext Ultra workflows include an enzyme mixture to perform end repair for 30 min at 20 °C, followed by 3′ A-tailing for 30 min at 65 °C.

We applied our approach to library construction of human genomic DNA for sequencing on an Illumina MiSeq and compared the sequencing results to libraries generated using the soluble enzyme mixture PKT or from three commercially available kits. To avoid the complication of potential amplification-derived bias, PCR-free libraries were used (Fig. 4). Indeed, the method using immobilized enzymes for end repair and 3′ A-tailing notably improves sequence coverage of the AT-rich regions in the human DNA libraries in comparison to the method using the PKT enzyme mixture with a high temperature incubation step following the end repair reaction (Fig. 4a). The libraries produced by the commercially available kits all yielded significant bias with notable under-representation of AT rich DNA (Fig. 4b). Similar to the method using PKT, the protocols of NEBNext Ultra II DNA Library Prep Kit and Kapa Hyper Prep Kit adopt high temperature incubation at 65 °C (Fig. 1c). The protocol of Illumina TruSeq DNA PCR-free LT Library Preparation Kit includes a 5 min incubation at 70 °C following 3′ A-tailing for 30 min at 37 °C (Fig. 1d). In addition, use of the Illumina protocol resulted in a reduced library yield (Supplementary Fig. 9), probably due to a substantial loss of DNA from the bead purification step after the end repair reaction. Similar to the end repair enzyme mixtures of the three commercial kits the PKT mixture uses end repair enzymes, i.e. T4 DNA pol and T4 PNK. However, the PKT mix resembles the streamlined Kapa Hyper Prep Kit and NEBNext Ultra II DNA Prep Kits because it utilizes a thermophilic polymerase such as Taq DNA pol for 3′ A-tailing whereas the Illumina TruSeq kit uses a mesophilic polymerase such as exonuclease-deficient Klenow Fragment. Despite the difference in 3′ A-tailing enzymes, the Illumina TruSeq protocol still displayed significant GC-bias in sequence coverage. The library yield was relatively low when the end repair reaction was catalyzed at 20 °C by immobilized enzymes; however, the yield was significantly increased by performing end repair at 37 °C with immobilized enzymes (Supplementary Fig. 9). Though the major metrics used in evaluating NGS data from different methods are mostly comparable (Supplementary Fig. 10), the immobilized enzyme method, however, produced a slightly higher percentage of chimeric reads than the method of soluble enzymes probably due to less efficient end repair at 20 °C or 3′ A-tailing at 37 °C.

### High temperature treatment as a primary source of GC-bias

**Figure 5:**
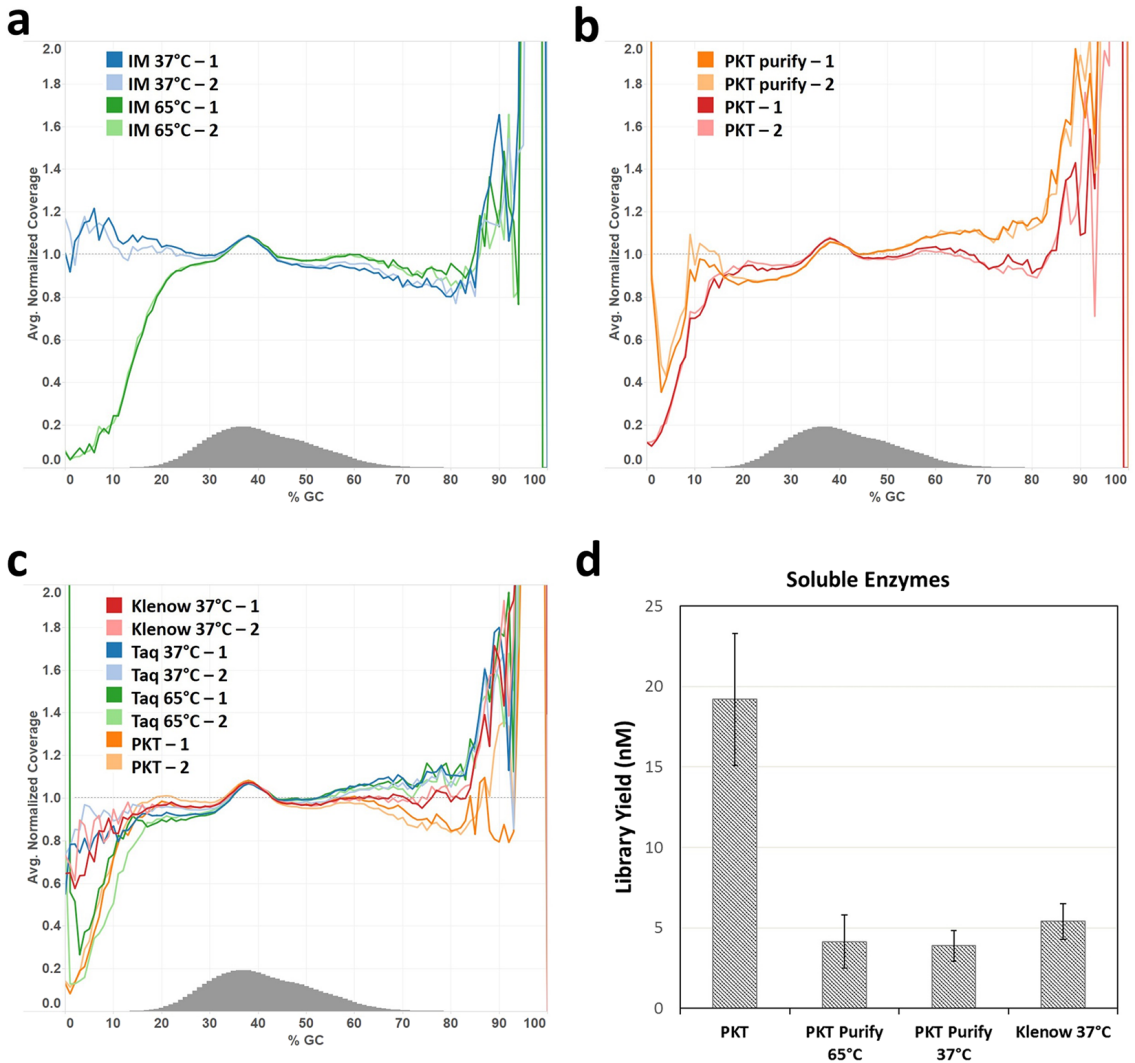
Effect of end repair and 3′ A-tailing at high temperature on GC-bias in Illumina reads from PCR-free human DNA libraries. (**a**) Comparison of GC-bias curves in duplicate libraries prepared by immobilized enzymes with 3′ A-tailing performed at 37 °C (IM 37 °C -1 and IM 37 °C -2, in blue) or 65 °C (IM 65 °C -1 and IM 65 °C -2, in green) revealed a dramatic effect of 3′ A-tailing at high temperature on sequence coverage of the AT-rich regions from human DNA libraries. (**b**) GC-bias curves were generated from two sets of duplicate libraries produced using the soluble enzyme mixture PKT with (PKT purify-1 and PKT purify-2) or without (PKT-1 and PKT-2) a purification step between end repair and high temperature incubation (with Taq DNA pol added to the samples following purification). Although some bias against AT-rich regions can be attributed to the end repair step, the elevated temperature contributes to the majority of the dropouts in the AT-rich regions. (**c**) Shown are the GC-bias curves from 4 sets of duplicate libraries produced by the method of soluble enzymes. Two sets of the duplicate libraries were purified after end repair with PK mixture and then treated at 37 °C with Klenow Fragment (3′-5′ exo^−^) (red, Klenow 37 °C-1 and Klenow 37 °C-2) or Taq DNA pol (blue, Taq 37 °C-1 and Taq 37 °C-2). The other two duplicate sets were prepared using the high temperature treatment protocol either with (green, Taq 65 °C-1 and Taq 65 °C-2) or without (orange, PKT-1 and PKT-2) a purification step between end repair with PKT and treatment with Taq DNA pol at 65 °C for 30 min. (**d**) Comparison of library yield of the three sets described above with or without (PKT on the left) a purification step between end repair and 3′ A-tailing indicates that purification caused substantial loss of library DNA.

The key difference between the different methods validated is the lack of high temperature incubation using immobilized enzymes, implying that incubation at an elevated temperature is a major cause of bias against the AT-rich fraction in a human DNA library. We examined the libraries prepared by several modified protocols. A lower sequence coverage in the high AT-content regions of the human genome was observed when the 3′ A-tailing step was carried out at 65 °C instead of 37 °C using the immobilized enzyme method (Fig. 5a). Next, inclusion of a purification step between end repair and high temperature incubation in the soluble enzyme workflow (PKT) only modestly reduced the GC-bias against the AT-rich fraction (Fig. 5b). In addition, a combination of purification after end repair at 20 °C and 3′ A-tailing at 37 °C substantially reduced the bias in the libraries using the soluble enzyme method (Fig. 5c). However, these purification steps consistently resulted in lower library yields (Fig. 5d).

### FFPE DNA library construction

The data presented demonstrate that the immobilized enzymes protocol can be applied to ideal genomic samples with plenty of material recently extracted. To further assess the utility of the immobilized enzymes method on more challenging samples, we have performed Illumina DNA library construction using human FFPE DNA with or without DNA repair as described previously^18^. We used FFPE DNA samples as an example of challenging genomic DNA for which library preparation has been shown to be problematic and DNA has been shown to be extensively damaged, notably cytosine deamination^19^.

Additionally, previous studies have demonstrated the utility of repairing the FFPE DNA prior to library preparation on both the library yield and sequencing accuracy^18^. We therefore would like to assess whether immobilized enzymes can lead to a good quality library with challenging samples and whether our immobilized enzyme protocol is compatible with DNA repair. Towards this goal, we prepared libraries using the same FFPE kidney tumor genomic DNA with or without DNA repair following NEBNext Ultra II DNA library workflow or immobilized enzyme workflow as described in the methods, respectively.

The data shows that the immobilized enzyme method improved sequence coverage in the extremely AT-rich regions of the FFPE DNA although significant GC-bias was still detected for the FFPE libraries (Supplementary Fig. 11). Furthermore, the immobilized enzyme method resulted in improved library yields when libraries were treated with an additional DNA repair step compared to those without a DNA repair step during the library construction (Supplementary Fig. 12). The library yield from the immobilized enzyme method is slightly lower probably due to a lower efficiency in end repair reaction being carried out at 20 °C. Furthermore, we observed a decrease of the C to T mutation rate in the repaired samples indicating that the immobilized enzyme protocol is compatible with the repair step (Supplementary Fig. 13).

## Discussion

**Figure 6:**
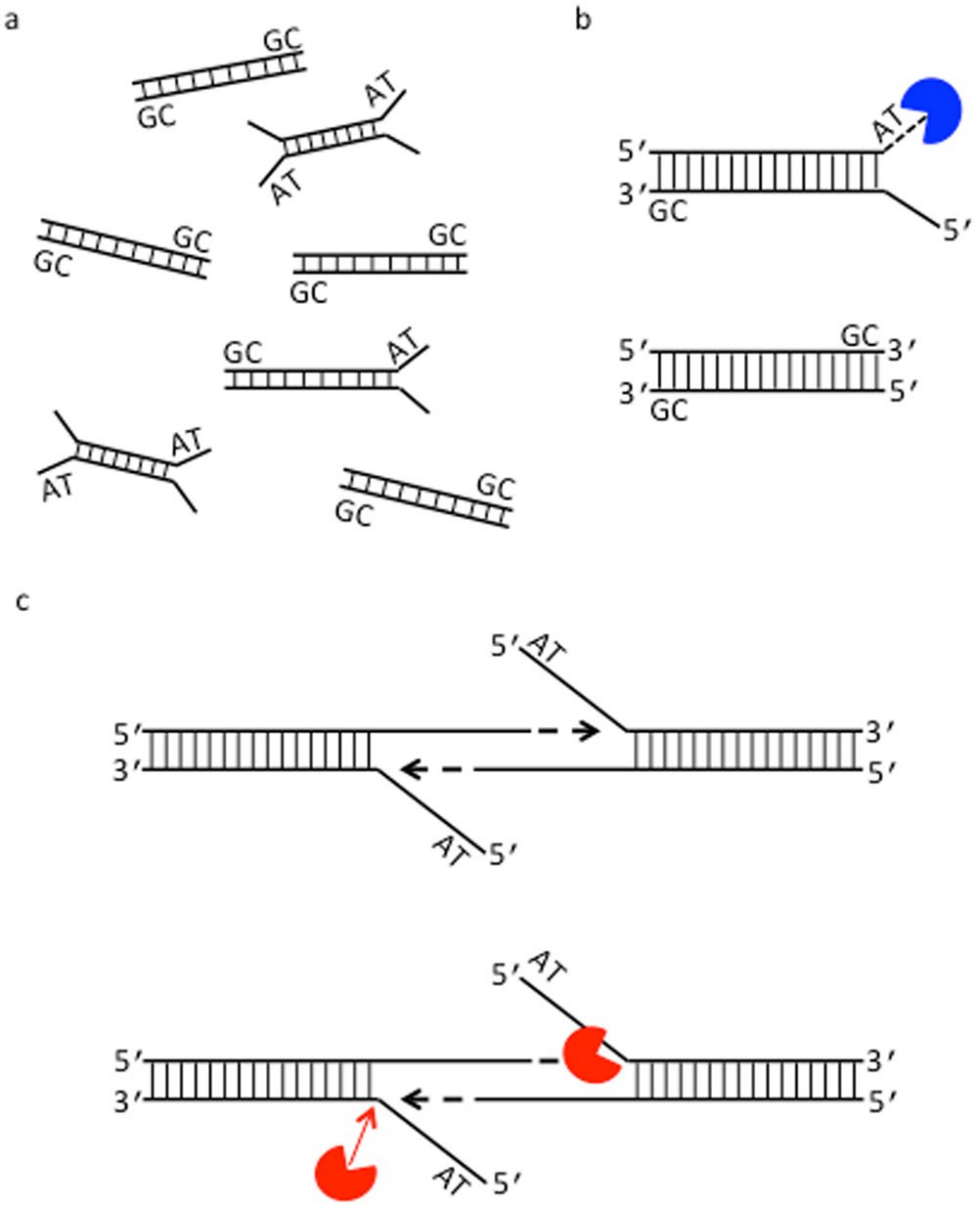
A model for GC-related sequence coverage bias in amplification-free NGS data. (**a**) A schematic of DNA end “breathing” (or “fraying”) present in the AT-rich fraction of a DNA library. DNA thermal breathing refers to spontaneous local conformational fluctuations, leading to unpaired bases at the ends of DNA duplex. The extent of breathing is highly dependent upon temperature and DNA sequence so that AT-rich segments (AT) melt before GC-rich segments (GC). The difference of the end breathing profile relevant to GC-content leads to less efficient end-polishing of AT-rich fragments during library construction using DNA modifying enzymes, resulting in the under-representation of the AT-rich regions. (**b**) Degradation of AT-rich DNA by 3′-5′ exonuclease activity of T4 DNA pol (blue). Preferential degradation of AT-rich DNA fragments that undergo terminal base pair breathing may occur at the end repair step or during high temperature incubation. (**c**) Processing AT-rich DNA by Taq DNA pol at elevated temperatures. During high temperature incubation, for example, at 65 °C or 70 °C, the ends of AT-rich DNA fragments melt into transient or predominant single-stranded structures. Taq DNA pol (red) can act on these DNA substrates by its polymerization and 5′ nuclease activities as previously described^34^, yielding unintended cleavage and primer extension products. Arrow (red) indicates the position of cleavage whereas arrow in black indicates the orientation of primer extension due to intermolecular annealing of two single-stranded 3′ terminal sequences. Primer extension may also occur from intramolecular annealing of a single-stranded 3′ terminal sequence.

High yield, low bias sample preparation protocols are of particular importance for DNA sequencing applications to ensure that the sequencing data accurately reflects the input sample. Effort has been made in selecting enzymes for library preparation, as well as in optimizing their ratios and overall workflows to achieve high efficiency and specificity^6,20,21^. One of the critical tasks comprises the identification of the major factors that generate uneven sequencing coverage and the exploration of new solutions to correct these biases^22,23^. In this study, we applied a stepwise approach to the identification of the sources of sequence coverage bias against the AT-rich fraction in a human genomic library. Our examination of the major enzymatic steps using synthetic DNA substrates, possessing various GC content and terminal structures, suggests that end repair and high temperature incubation during A-tailing are the primary sources that contribute to the under-representation of the high AT-content DNA fraction in human DNA libraries using widely-used NGS library construction protocols. The data support our hypothesis that DNA thermal breathing causes depletion of the AT-rich fraction in human DNA library at elevated temperatures due to nuclease and polymerase activities of the DNA modifying enzymes used in library construction as depicted in Fig. 6. Based on these findings, we succeeded in significant reduction of this sequence coverage bias by employing an immobilized enzyme workflow to avoid high temperature treatment of DNA library.

We have observed preferential degradation of AT-rich DNA fragments, presumably exposed by terminal thermal breathing, under the conditions for end repair and 3′ A-tailing at elevated temperatures. DNA thermal breathing is defined as the transient denaturation of nucleic acid base pairs because of thermal fluctuations. Partially open base pairs at fragment ends may comprise a significant fraction of the AT-rich sequences in a library pool, even at temperatures well below the melting temperature of the DNA duplex^24^. Previous studies have shown that terminal base composition can drastically affect the activity of DNA modifying enzymes, due to DNA terminal breathing^25^. It has been shown that terminal A-T base pairs can fluctuate between paired and unpaired states much more frequently than G-C base pairs^26^. It has been proposed that palindromic sequence artifacts identified during NGS sample preparation of historic and ancient DNA may be caused by degradation-specific processes due to the formation of single-stranded DNA by the high AT-content terminal sequences^12^. Thus terminal breathing of AT-rich fragments is likely accounted for exonucleolytic degradation by T4 DNA pol, leading to lower sequence coverage. The extent of exonuclease-mediated degradation would be highly dependent upon the reaction components, including enzyme units, reaction temperature and the GC-content of DNA.

The ultimate success of Illumina DNA library preparation is strongly dependent on efficient 3′ A-tailing for adaptor ligation. Our work establishes that high temperature incubation during 3′ A-tailing as the most influential factor in producing lower sequencing coverage in high AT-content regions. Removal of end repair enzymes did not eliminate the bias in sequence coverage against the AT-rich fraction if 3′ A-tailing was performed at 65 °C for both soluble and immobilized enzyme methods. A previous study showed that 3′ terminal residue affected 3′ A-tailing by Taq DNA pol^27^. Our work, however, reveals no significant difference in 3′ A-tailing efficiency between the synthetic AT-rich and GC-rich DNA substrates in the presence of sufficient amount of Taq DNA pol despite that the reaction rate is higher for the GC-rich substrate compared to the AT-rich substrate tested. The data support that once DNA end breathes open the single-stranded 3′ sequences of the AT-rich DNA can participate in intramolecular or intermolecular annealing to form templates for Taq DNA pol. Taq DNA pol are bifunctional molecules having both polymerization and 5′ nuclease activities, and can act on these templates for primer extension and cleavage (Fig. 6), resulting in unligatable products in DNA library or unmapped sequences in NGS.

We demonstrated an effective solution to this GC-bias problem by employing an enzyme immobilization strategy as an alternative workflow. We successfully engineered immobilized DNA modifying enzymes for end repair and 3′ A-tailing by covalent conjugation to magnetic beads. This approach enables solid phase enzymatic catalysis by DNA blunting and phosphorylation steps in library construction and convenient removal of enzymes upon the completion of enzymatic reactions. A-tailing is accomplished by Taq DNA pol at 37 °C, rather than an elevated temperature often called for when using this thermophilic enzyme. The NGS libraries prepared by this novel workflow displayed a drastically improved sequence coverage in the high AT-content regions in an amplification-free human DNA library. This method allows fast removal of enzymes without a significant reduction in library yield whereas other bead based methods for enzyme removal are more time-consuming, result in lower library yield, and do not improve bias. The immobilized enzymes method yields a slightly higher percentage of chimeric reads compared to the commercially available kits; this may be caused by lower A-tailing activity resulting in higher blunt-end ligation of DNA fragments in the libraries.

The method presented here exhibits significantly lower bias in sequencing coverage of high AT-content regions and may potentially improve the quality of NGS and data interpretation. The importance of AT-rich regions is well documented and has yet to be explored further for research and diagnostic purpose. For example, AT-rich sequences are concentrated in the nucleosome-free regions associated with transcription start sites of most genes and are thought to facilitate the removal of nucleosomes by interacting with chromatin remodeling complex^28^. It has also been suggested that AT-rich repeats are responsible for translocations associated with human genome disorders and these elements are in hot spots of genome rearrangement during evolution^29^.

Furthermore, this technique exploits the DNA modifying enzymes required for the current Illumina library construction with a number of advantages including fast enzyme removal without the requirement of high temperature treatment or purification by AMPure beads. It is worth noting that Nextera technology employs *in vitro* transposition to prepare sequencer-ready libraries and requires no heat treatment. However, the GC-bias of a Nextera library has been found to be comparable to that observed with a physical shearing method^11,30^, probably owing to insertion site bias or amplification-generated bias.

This study shows that the soluble enzyme components in single-tube enzyme formulation can affect end repair activities of T4 DNA pol. It is conceivable that the enzyme immobilization approach may offer additional advantages in DNA manipulation using multi-enzyme catalysis, including preventing simultaneous binding of multiple enzyme molecules on a DNA substrate and enabling single-molecule-catalyzed reactions in a manner unachievable by their soluble forms.

Preliminary results on FFPE samples indicate that immobilized enzymes can improve the current protocol for sequencing challenging and damaged samples. Whether sequencing FFPE or ancient DNA samples is suitable using immobilized enzymes protocol would require further experiments and analysis that is beyond the scope of this study. The immobilized form presumably carries an additional cost of beads to perform enzyme conjugation. Furthermore, successful implementation of enzyme immobilization technology requires innovative solutions to overcome potential challenges such as lower specific activity, longer incubation time and additional equipment needs. With high-throughput sequencing already being applied in clinical contexts^31^, there is a pressing need for coupled multi-reaction workflows and automated production of sequencing libraries to produce high quality data. Our recent work on improvement of enzymatic activity and engineering of solid support materials may lead to the use of solid phase catalysis in automated sample preparation applications using robotics or microfluidic devices^17^.

## Methods

### Materials

All DNA modifying enzymes and NEBNext Ultra II Library Prep Kit were provided by New England Biolabs (NEB) (Ipswich, MA). Illumina TruSeq DNA PCR-Free LT Library Preparation Kit was obtained from Illumina (San Diego, CA) and Kapa Hyper Prep Kit was obtained from Kapa Biosystems (Wilmington, MA). Human DNA was obtained from Promega (Madison, WI). DNA oligos were synthesized by Integrated DNA Technologies (Coralville, IA) and dissolved in nuclease-free water prior to use (Supplementary Table 1). Oligo pairs were used to generate double-stranded DNA substrates containing multiple G-C or A-T pairs at their termini (Fig. 2a). Each pair of complementary oligos was mixed to 5 µM final concentrations and annealed by incubation at 80 °C for 5 min in 1x NEB Buffer 2 (NEB) and slowly cooled at room temperature. For instance, 47-AT and 47-GC were produced by annealing a 47 nt 5′ FAM-labeled oligo to a 51 nt complementary oligo to form a 3′ recessed duplex, containing multiple terminal A-T and G-C base pairs, respectively. In addition, to monitor the fate of both strands, a 5′ FAM-labeled oligo was used to anneal with its complementary oligo labeled with Rhodamine X (ROX) at its 3′ end to form a DNA duplex. A 25 bp blunt-end DNA substrate with a 3′ FAM labeled oligomer was used to analyze 5′ phosphorylation activity of T4 PNK. The complementary oligos are 5′-GTCCTGTGTGAAATTGTTATCCGCT-3′ and 5′-AGCGGATAACAATTTCACACAGGAC-3′ FAM. pSNAP_f_-tag(T7), a derivative of pSNAP-tag(T7)-2 (NEB), was used to subclone the gene encoding T4 DNA polymerase, T4 polynucleotide kinase and Taq DNA polymerase. Each protein coding sequence was fused in-frame to the 3′ end of SNAP_f_, using Sbf I and Not I sites, and a 6-histidine tag fused to the 5′ end of SNAP_f_ in pSNAP_f_-tag (T7). The resulting plasmids were used for expression of the fusion proteins in *E. coli* strain T7 Express (NEB) and subsequent affinity purification by Ni-NTA agarose (Qiagen, Germany). The purified SNAP-tagged enzyme sample was then dialyzed into a buffer containing 50% glycerol for long-term storage at −80 °C.

### Protein immobilization

SNAP-tagged enzymes were conjugated to benzylguanine (BG)-functionalized magnetic beads (NEB) by mixing 100 μg of protein with 100 μl bed volume of beads suspended in 500 µl of phosphate-buffered saline (PBS) at 4 °C overnight as described previously^17^. The conjugated sample was washed a total of 8 times using 1 ml of phosphate buffered saline (PBS). The immobilized enzymes were dialyzed into the same buffer used for the soluble form containing 50% glycerol and stored at −20 °C.

### Capillary Gel Electrophoresis Analysis

Enzyme modification of the DNA substrates was monitored using fluorescence capillary gel electrophoresis (CE) as described previously^32^. A typical exonuclease assay contains a double-stranded DNA substrate end-labeled with a FAM or ROX (Supplemental Table 1) 1.5 units of T4 DNA polymerase (NEB) in a 10 µl reaction in the presence of 1x NEBNext End Repair Reaction Buffer (NEB) containing 200 µM of each of dNTPs. The reactions were performed in a T-100 Thermocycler (Bio-Rad Laboratories, Hercules, CA). Enzyme mix PKT was comprised of approximately 1,200 units/ml T4 DNA polymerase, 2,000 units/ml T4 PNK and 2,000 units/ml Taq DNA polymerase (NEB) while PK contained T4 DNA polymerase and T4 PNK only. Assays for 3′ A-tailing activity were carried out at 37 °C or 65 °C using annealed oligonucleotides in a final concentration of 0.5 µM and 2 units of Taq DNA pol, Klenow Fragment (3′-5′ exo^−^) or Hemo KlenTaq (NEB) in 10 µl reaction in the presence of 1x NEBNext End Repair Buffer (NEB) or a 3′ A tailing buffer. NEBNext dA-Tailing Reaction Buffer (dAT) contains 10 mM Tris-HCl, 10 mM MgCl_2_, 50 mM NaCl,1 mM DTT 0.2 mM dATP, pH 7.9 @ 25 °C, with dAQ buffer containing a supplement of 7% polyethylene glycol (PEG) 6000 (Sigma). The reactions were terminated by diluting in 50 mM EDTA and 0.1% Tween-20. The percent degradation of FAM-labeled (or ROX-labeled) oligonucleotide was determined by analyzing the CE data using Peak Scanner software. Triplicate samples were quantified and error bars were added to indicate one standard deviation.

### DNA library preparation

Human genomic DNA (Mix) from Promega (Fitchburg, WI) was diluted to a final concentration of 100 µg/ml in 10 mM Tris-HCl, 1 mM EDTA, pH 7.5, and fragmented to 200 base pairs (bp) using a Covaris AFA S2 system (Covaris, Woburn, MA). The setting was 5% duty cycle, intensity 10, and 200 cycles per burst for 6 minutes. PCR-free libraries were made using 1 µg of fragmented genomic DNA and the NEBNext Ultra Library Prep Kit or Kapa Hyper Prep kit, or the PKT mix, all of which conduct end repair at 20 °C for 30 min followed by incubation at 65 °C for 30 min for 3′ A-tailing. The protocol was further modified by addition of a purification step for enzyme removal after end repair, followed by 3′ A-tailing for 30 min at 37 °C or 65 °C. Human DNA libraries were also prepared using Illumina TruSeq DNA PCR-free LT Library Preparation Kit, which carries out end repair at 30 °C for 30 min, followed by a purification step for clean up and size selection, A-tailing at 37 °C for 30 min, and heat treatment for 5 min at 70 °C. For the immobilized enzyme protocol, 1 µg of sheared DNA was end-repaired for 30 min at 20 °C (or 37 °C) using immobilized T4 DNA pol and T4 PNK in 1x NEBNext End Repair Buffer. The end repair enzymes were pelleted by magnet and the supernatant was transferred to a new tube for 3′ A-tailing in dAQ buffer for 30 min at 37 °C (or 65 °C) with immobilized Taq DNA pol. Each library was ligated to preannealed full-length paired-end Illumina adaptors from the TruSeq DNA PCR-free Library Prep Kit. DNA libraries were size-selected and analyzed to determine the size distribution using an Agilent High Sensitivity DNA Kit on a Bioanalyzer 2100 (Agilent Technologies, Santa Clara, CA). Library yields were further determined by qPCR using the NEBNext library quant kit for Illumina.

FFPE kidney tumor genomic DNA (BioChain Institute Inc., Newark, CA) libraries were prepared with or without DNA repair following NEBNext Ultra II DNA library or immobilized enzyme workflows as described above. For DNA repair, prior to the end repair step, the fragmented DNA (15 ng) was treated for 15 minutes at 20 °C with NEBNext FFPE DNA Repair Mix (NEB). All other steps in the library preparation protocol were kept the same as for the unrepaired DNA library construction. All libraries were indexed and amplified with 8 cycles of PCR, and paired-end sequenced on an Illumina MiSeq platform.

The libraries were sequenced on an Illumina MiSeq in paired-end mode (2 × 75 bp). Reads were adapter trimmed (SeqPrep, v1.1 https://github.com/jstjohn/SeqPrep) before alignment to the GRCh38 reference genome (from ftp://ftp.ncbi.nlm.nih.gov/genomes/all/GCA/000/001/405/GCA_000001405.15_GRCh38/seqs_for_alignment_pipelines.ucsc_ids/GCA_000001405.15_GRCh38_no_alt_plus_hs38d1_analysis_set.fna.gz) (bowtie 2.3.2, end-to-end, -X 1000)^33^. GC Bias was assessed using Picard’s CollectGCBiasMetrics (Picard 2.7.1). Relevant Low-GC regions were identified by intersecting 100 bp windows (bedtools v2.25.0) having GC fraction <0.2 with 80% overlap with features in the Gencode v26 basic genes. Coverage of low GC regions was assessed using bedtools cov.

## Electronic supplementary material

Supplementary Information

## Data Availability

The data supporting the findings of this study are available within the paper and its Supplementary Information files are available from the corresponding author upon request.
