# Supplementary material for: Solid-phase enzyme catalysis of DNA end repair and 3′ A-tailing reduces GC-bias in next-generation sequencing of human genomic DNA
Source: Sci Rep. 2018 Oct 26;8:15887. doi: 10.1038/s41598-018-34079-2 (PMC6203771; doi:10.1038/s41598-018-34079-2)
Supplement: Supplementary file 1 — Supplementary Information [file 41598_2018_34079_MOESM1_ESM.pdf]

Supplementary Information

Solid-phase enzyme catalysis of DNA end repair and 3′ A-tailing reduces GC-bias in next-generation sequencing of human genomic DNA

Aihua Zhang, Shaohua Li, Lynne Apone, Xiaoli Sun, Lixin Chen, Laurence M. Ettwiller, Bradley W. Langhorst, Christopher J. Noren, Ming-Qun Xu*

New England Biolabs, Inc., 240 County Road, Ipswich, MA 01938, USA

*Correspondence and requests for materials should be addressed to M.X. (email: xum@neb.com)

Supplementary Table 1. DNA substrates and oligonucleotide sequences

Name	Sequence
51GC-1FW	5′-FAM-CGAATTTATGCCTGCAGGCGCCGAATATTGCCTGAGCTATCGCTGCGGCGC-3′
51GC-1RV	5′p-GCGCCGCAGCGATAGCTCAGGCAATATTCGGCGCCTGCAGGCATAAATTCG-3′
51AT-1FW	5′-FAM-CGAATTTATGCCTGCAGGCGCCGAATATTGCCTGAGCTATCATTTAATATA-3′
51AT-1RV	5′p-TATATTAAATGATAGCTCAGGCAATATTCGGCGCCTGCAGGCATAAATTCG-3′
51-AT65%	5′-FAM-TAAATTTATTCCTGCAGGCGCCGAATATTGCCTGAGCTATCATTTAATATA-3′
51-AT65% RV	5′-TATATTAAATGATAGCTCAGGCAATATTCGGCGCCTGCAGGAATAAATTTA-3′
51AT90%	5′-FAM-TAAATTTATTAATACAATCTTCTAATATTGATGATTATATTATTTAATATA-3′
51AT90% RV	5′-TATATTAAATAATATAATCATCAATATTAGAAGATTGTATTAATAAATTTA-3′
51-GC	5′-FAM-CGCGTTTATGCCTGCAGGCGCCGAATATTGCCTGAGCTATCGCTGCGGCGC-3′
47-GC	5′-FAM-CGCGTTTATGCCTGCAGGCGCCGAATATTGCCTGAGCTATCGCTGCG-3′
54-GC	5′-FAM-CGCGTTTATGCCTGCAGGCGCCGAATATTGCCTGAGCTATCGCTGCGGCGCATA-3′
51-GC RV	5′-GCGCCGCAGCGATAGCTCAGGCAATATTCGGCGCCTGCAGGCATAAACGCG-3′
51-AT	5′-FAM-CGCGTTTATGCCTGCAGGCGCCGAATATTGCCTGAGCTATCATTTAATATA-3′
47-AT	5′-FAM-CGCGTTTATGCCTGCAGGCGCCGAATATTGCCTGAGCTATCATTTAA-3′
54-AT	5′-FAM-CGCGTTTATGCCTGCAGGCGCCGAATATTGCCTGAGCTATCATTTAATATAGCG-3′
51-AT RV	5′-TATATTAAATGATAGCTCAGGCAATATTCGGCGCCTGCAGGCATAAACGCG-3′
60GC-AT	5′-FAM-CGAATTTATGCCTGCAGGCGCCGAATATTGCCTGAGCTATCGCTGCGGCGCTTTAATATA-3′
60GC-AT RV	5′-TATATTAAAGCGCCGCAGCGATAGCTCAGGCAATATTCGGCGCCTGCAGGCATAAATTCG-Rox-3′

Oligonucleotide pairs were used to generate double-stranded DNA substrates by annealing a 5′ FAM labeled oligo with a complementary oligo as depicted in Fig. 2. To monitor the fate of both strands, a complementary oligo, for example, 51AT90% RV was end-labeled with 3′ ROX to form a duplex with 5′ FAM-labeled 51AT90%

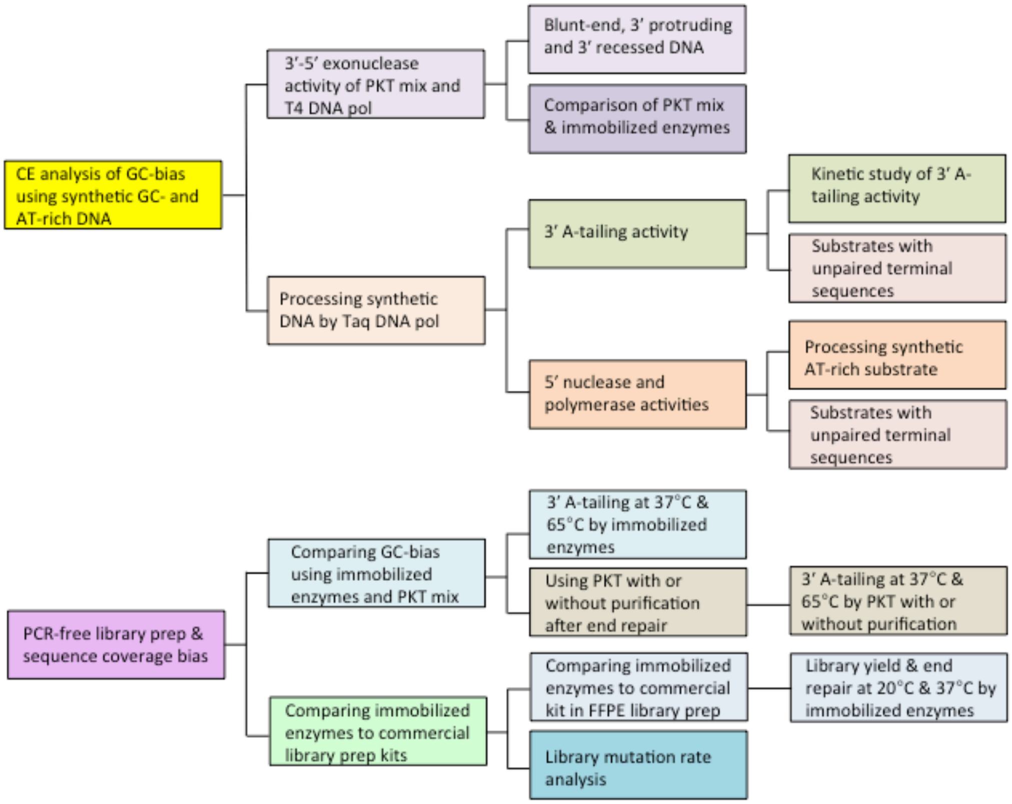

Supplementary Figure 1. Framework for study of GC-bias in library construction and development of immobilized enzyme method.

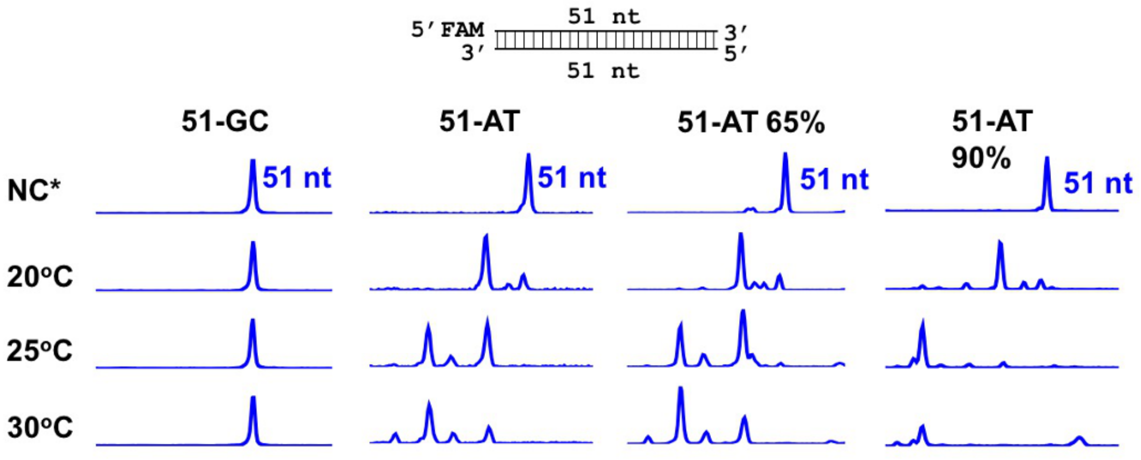

Supplementary Figure 2. CE analysis of exonuclease activity of end repair mixture PKT using synthetic DNA substrates with various AT-content. Each blunt-end DNA duplex contains a 5′ FAM-labeled 51 nucleotide (nt) oligomer possessing either a GC-rich 3′ terminus (51-GC) or an AT-rich 3′ terminus annealed to a complementary oligomer (Supplementary Table 1). The substrates were incubated at various temperatures for 30 min with PKT in an end repair buffer (containing all four dNTPs), followed by CE and Peak Scanner software analysis. The data reveal exonuclease-mediated degradation in a temperature-dependent manner in all three substrates possessing multiple terminal A-T pairs, 51-AT (59%AT), 51-AT 65% (65% AT content), and 51-AT 90% (90% AT content), yielding a loss of the FAM-labeled 51 nt peak and the appearance of multiple peaks corresponding to smaller degradation species, when compared to the 51 nt peak in the negative control samples (NC*) in the absence of enzyme. However, treatment of the 51-GC with 3′ GC-rich terminal sequence resulted in no degradation of the 51 nt template.

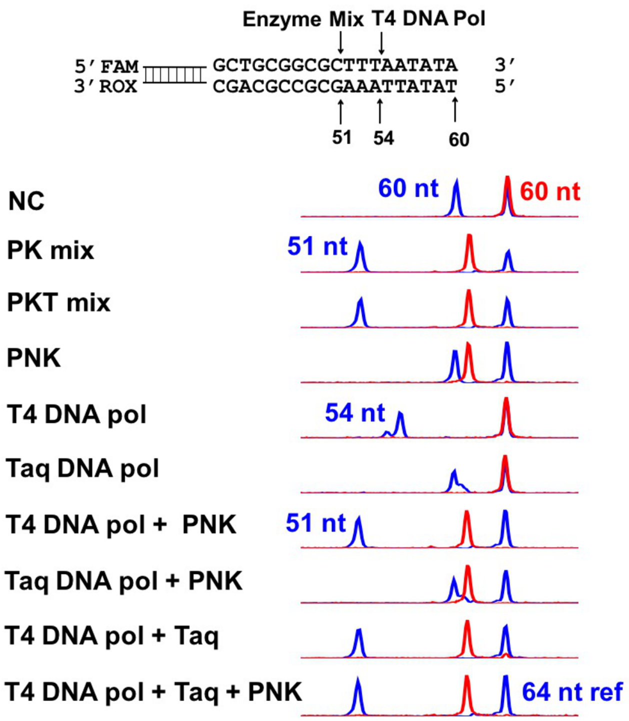

Supplementary Figure 3. Characterization of exonuceolytic activity of end repair enzymes and mixtures. Two complementary 60-nt oligomers were annealed to form a blunt-end DNA substrate possessing an AT-rich terminus preceded by a GC-rich segment (top panel). The oligos were synthesized with 5′ FAM (top strand) and 3′ ROX (bottom strand), in order to monitor the fate of both strands in the duplex. The strongly annealing GC-rich region is included to block further degradation of DNA 5′ of the AT-rich terminal sequence by 3′-5′ exonuclease activity. The substrate was incubated at 37°C for 30 min with an end repair enzyme mixture or T4 DNA pol supplemented with PNK or Taq DNA pol in a dNTP-containing end repair buffer. CE analysis reveals that the exonuclease activity of T4 DNA pol was enhanced in the enzyme mixture, by removing a shorter stretch of 3′ terminal sequence from the 5′ FAM-labeled template (blue), compared to the activity of a mixture (PK or PKT mix), compared to processing by T4 DNA pol alone. The arrows above the substrate sequence indicate the 3′ terminal positions of the FAM-labeled products observed in the assays with T4 DNA pol and different enzyme mixtures. The arrows below the sequence display the nucleotide positions of the full-length substrate and degradation products. The supplementation of PNK or Taq

DNA pol to T4 DNA pol elevated exonuclease activity, resulting in the removal of the entire 3′ AT-rich terminal sequence of the 5′ FAM-labeled oligomer, as observed for the enzyme mix reactions. Efficient 5′ Phosphorylation of the 3′ ROX-labeled oligomer (red) by PNK yielded a product completely shifted to the left side. A 64 nt reference (64-nt ref) oligo labeled with 5′ FAM was added to the samples for CE analysis after the reactions were terminated.

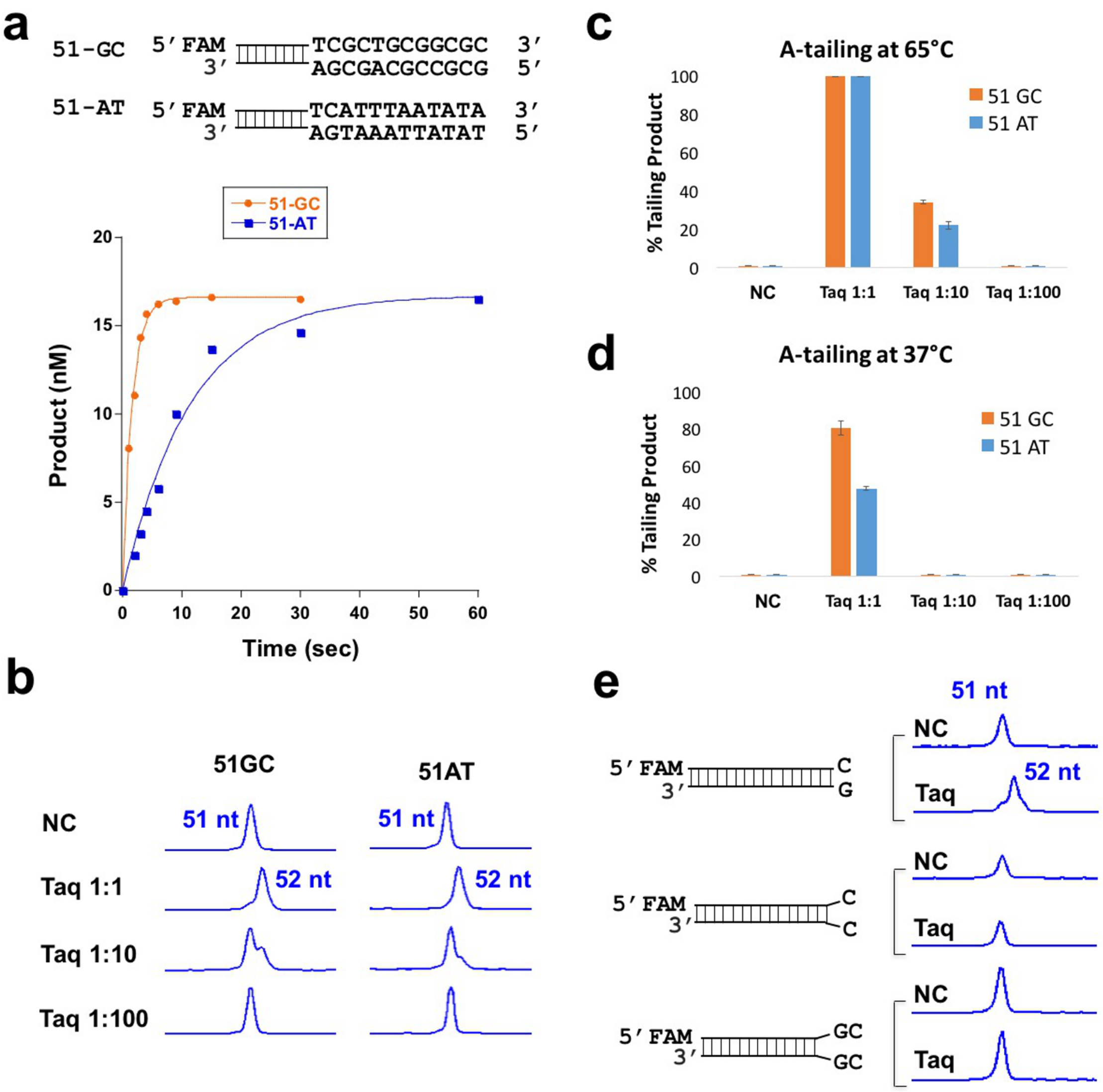

Supplementary Figure 4. Study of 3′ terminal transferase activity of Taq DNA pol. Enzymatic treatment was performed with 5′ FAM-labeled blunt-end DNA substrates 51-GC and 51-AT, possessing multiple terminal G-C and A-T base pairs, respectively. The substrates were incubated for 30 minutes at 37°C or 65°C in NEBNext dA-Tailing Reaction Buffer containing 200 μM of each dNTP for DNA synthesis. **a.** Kinetic study revealed different reaction rates at 65°C on 51-GC and 51-AT from 3′ terminal transferase (3′ A-tailing) activity of Taq DNA pol. **b.** Representative CE data indicate the efficiency of 3′ A-tailing reactions performed on 51-GC and 51-AT at 65°C in enzyme titration assays. The negative control (NC) reactions indicate the position of each 51-nt strand. **c.** 3′ A-tailing reactions of 51-GC

and 51-AT performed at 65°C using Taq DNA pol were analyzed by CE and quantified, indicating efficient A-tailing of both AT-rich and GC-rich DNA. **d**. CE analysis of 3′ A-tailing reactions of 51-GC and 51-AT performed at 37°C was quantified, showing lower A-tailing efficiency for the AT-rich substrate. **e**. Shown are the CE data for 3′ A-tailing activity of 51-GC and its derivatives carrying a single or double mismatched base pairs. The data indicates loss of A-tailing activity on the substrates possessing unpaired 3′ terminal sequences.

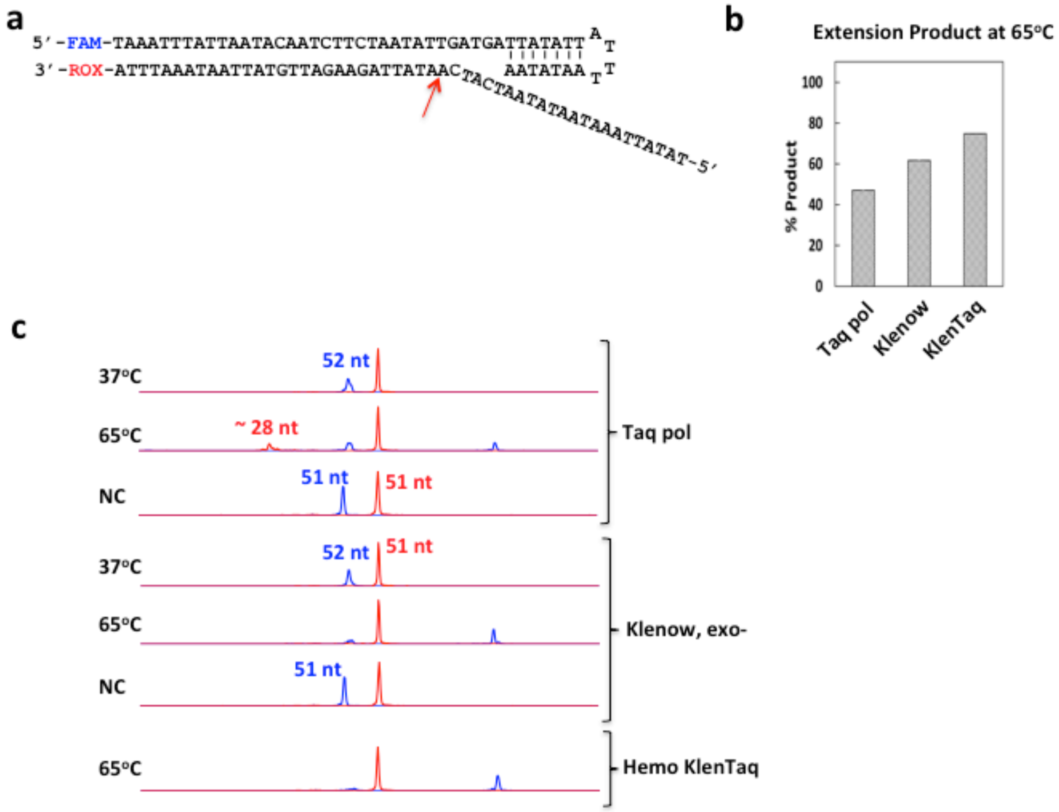

Supplementary Figure 5. Assays of 3′ A-tailing enzymes. a. A proposed structure for a synthetic AT-rich blunt-end DNA. The DNA duplex was produced by annealing of two complementary oligonucleotides, 51AT90% and 51AT90% RV end-labeled with 5′ FAM (blue) and 3′ ROX (red), respectively. Melting at the end of the DNA duplex may lead to intramolecular (shown) or intermolecular annealing between single-stranded 3′ terminal sequences, creating a template for primer

extension by a DNA polymerase. Arrow (red) indicates the approximately position of cleavage by the 5′-3′ flap endonuclease (5′ nuclease) activity of Taq DNA pol. 5′ FAM-labeled sequence shown includes a 3′ A addition. **b**. Efficiency of primer extension by various polymerases at 65°C revealed by CE data quantification. **c**. CE data from reactions performed at 37°C or 65°C using DNA polymerases, Taq DNA pol (Taq pol), Klenow Fragment (3′-5′ exo^-) and Hemo KlenTaq, a truncated version of Taq DNA pol lacking an nuclease domain. Incubation with Taq DNA pol (Taq pol) or Klenow Fragment (exo^-) at 37°C yielded the expected 3′ A-tailing product (52 nt, blue) and the 3′ ROX-labeled oligo probe (red) appeared stable. At 65°C Taq DNA pol, however, produced a large product of approximately 100 nt corresponding to primer extension activity on the 5′ FAM-labeled oligo (blue) and also a major product corresponding to cleavage of the 3′ ROX-labeled oligo (red) whereas Klenow Fragment (exo^-) and Hemo KlenTaq lacking nuclease activity yielded only the primer extension product (in similar size). The data support that at elevated temperatures DNA end breathing allows for DNA polymerase to act on AT-rich DNA templates via its polymerase and 5′ nuclease activities, yielding unintended products thereby causing under-representation of the AT-rich regions.

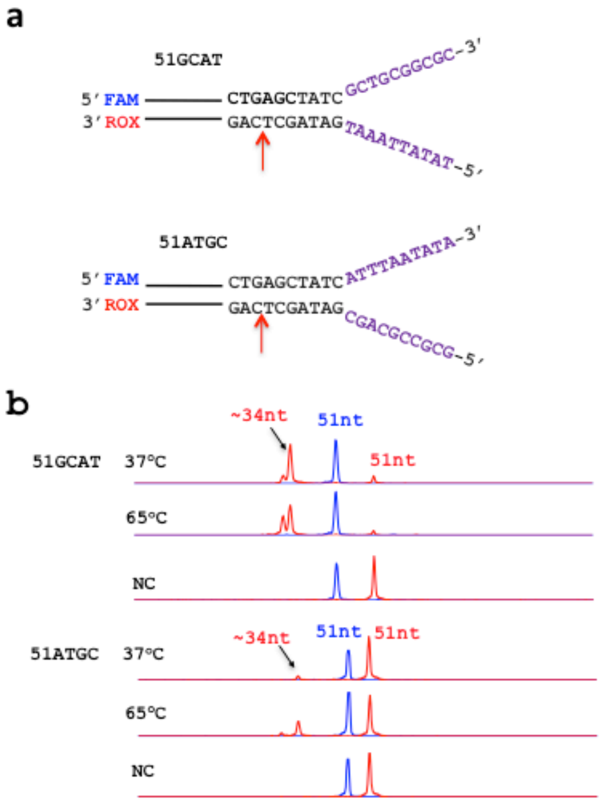

Supplementary Figure 6. Processing synthetic substrates possessing single-stranded terminal sequences by Taq DNA pol. a. DNA substrate 51GCAT was formed by pairing 51-GC with 51-AT RV, and 51ATGC was formed by pairing 51-AT with 51-GC RV (Supplementary Table 1). Both substrates were end-labeled with 5′ FAM (blue) and 3′ ROX (red) as shown for monitoring the fate of each strand, and contained unpaired terminal sequences. Intramolecular or intermolecular annealing of the single-stranded 3′ terminal sequences can create a template for DNA polymerase. Arrow (red) indicates the approximately position of cleavage by 5′ nuclease activity of Taq DNA pol as described below. **b**. Detection of 5′ nuclease activity of Taq DNA pol by CE analysis. Each substrate was incubated at 37°C or 65°C in the presence of Taq DNA pol and NEBNext End Repair Buffer containing all four dNTPs. For 51-GCAT, the ROX-labeled oligo was cleaved to yield a major product of approximately 34 nt at both 37°C and 65°C. For 51-ATGC, the ROX-labeled oligo was also cleaved to yield a similar product in size; however, the cleavage activity is much lower than that on 51-GCAT, in particular at 37°C. We reason that the higher

cleavage activity for 51GCAT substrate is likely due to its ability to form a stronger annealing template required for 5′ nuclease activity. The 5′ FAM-labeled oligo in each substrate appeared stable, and no 3′ A-tailing product (52 nt) nor primer extension product was detected, supporting that A-tailing activity of Taq DNA pol is dependent on base paired terminal structure.

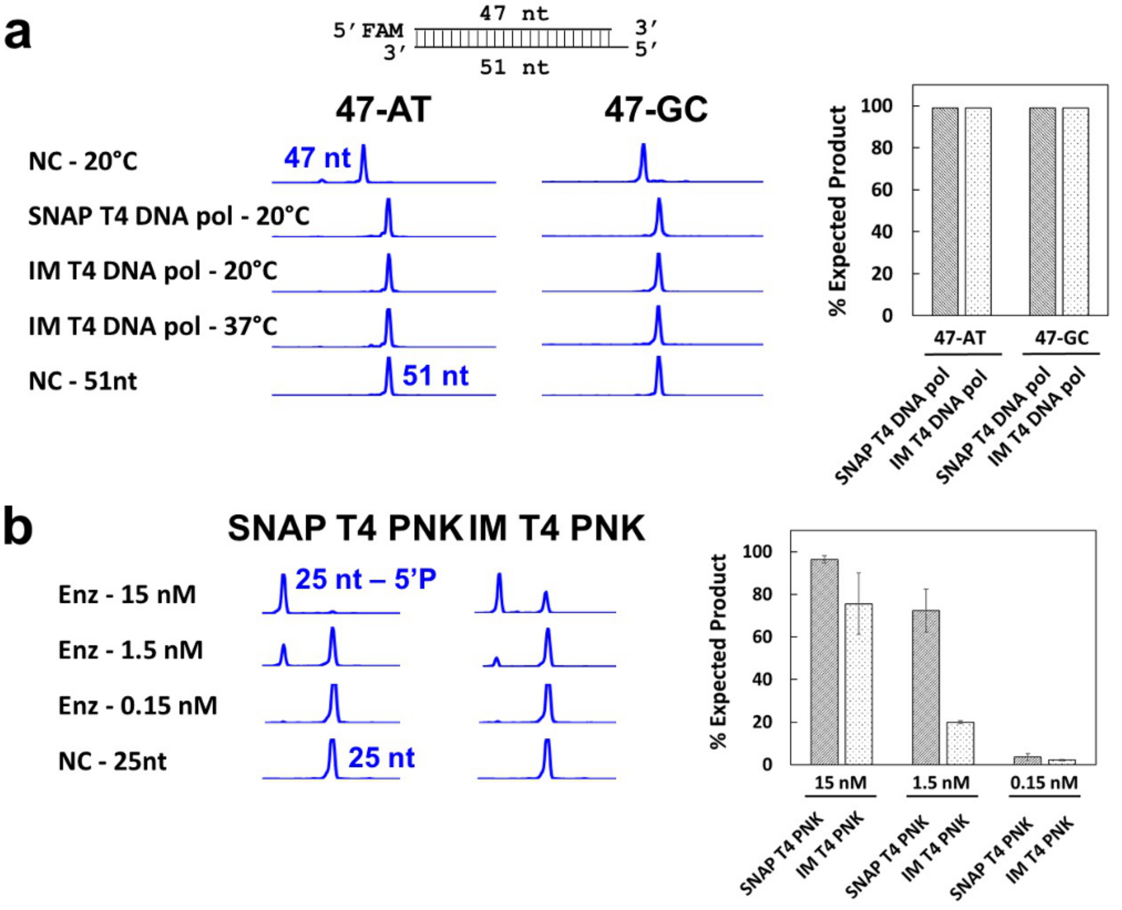

Supplementary Figure 7. Characterization of SNAP-tagged DNA modifying enzymes. a. CE analysis was conducted to monitor primer extension activity of soluble and immobilized SNAP-tagged T4 DNA pol on 3′ recessed double-stranded DNA substrates, 47-AT and 47-GC, with a 5′ FAM-labeled 47 nt oligo annealed to its complementary 51 nt oligo (51-AT RV and 51-GC RV, respectively) (left panels). The bar graph on the right shows the quantified data, indicating efficient fill-in reaction for both DNA templates containing multiple terminal A-T or G-C base pairs. **b**. Comparison of soluble and immobilized SNAP-tagged T4 PNK. A 25 bp blunt-end DNA substrate with a 3′ FAM labeled oligomer (25 nt) was used to analyze 5′ phosphorylation activity at various enzyme concentrations. 5′ Phosphorylation yielded a shift of the 5′ FAM-labeled 25 nt oligo to the left (shown as 25nt-5′P). The CE data were quantified and shown as a bar graph on the right.

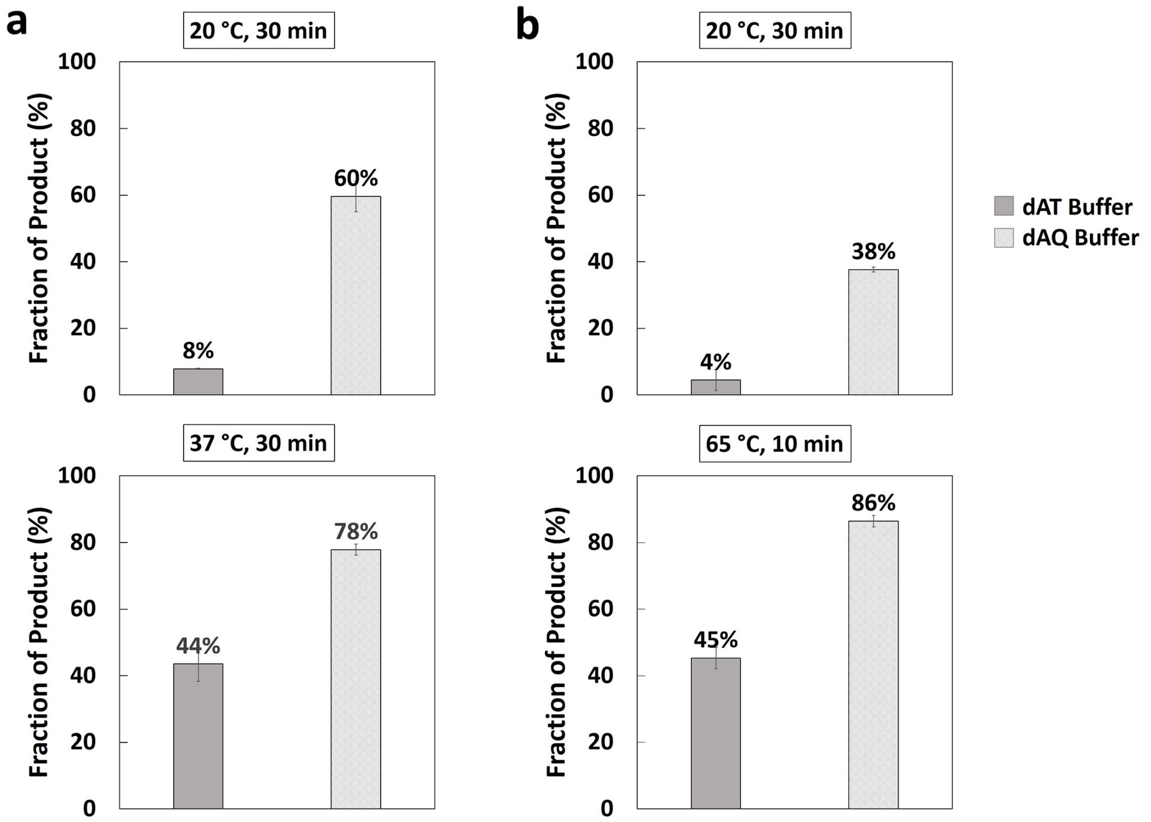

Supplementary Figure 8. Enhanced 3′ A-tailing activity of soluble and immobilized Taq DNA pol in the presence of polyethylene glycol (PEG). **a**. 3′ A-tailing activity at 20°C (top) or 37°C (bottom) for 30 min displayed by Taq DNA pol immobilized onto magnetic beads in buffer containing PEG (dAQ, dark shaded bars) or no PEG (dAT, light shaded bars). **b**. 3′ A-tailing activity of soluble Taq DNA Pol (NEB) at 20°C for 30 min (top) or 65°C for 10 min (bottom). 3′ A-tailing efficiency was measured by quantification of the CE data by PeakScan software. Both soluble and immobilized form exhibited higher 3′ A-tailing activity in the presence of PEG in 3′ A-tailing buffer.

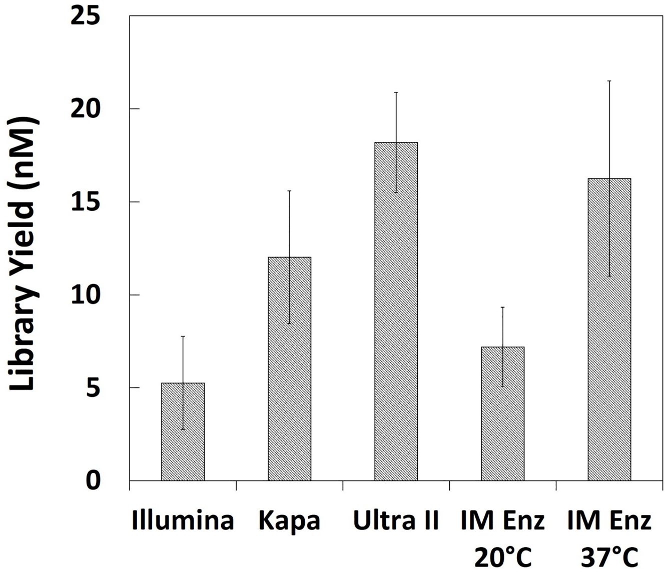

Supplementary Figure 9. Comparison of library yield. PCR-free human DNA libraries were prepared in duplicates using Illumina TruSeq DNA PCR-free LT Library Preparation Kit (Illumina), Kapa Hyper Prep Kit (Kapa) and NEBNext Ultra II DNA Library Prep Kit for Illumina (Ultra II), or immobilized enzymes (IM Enz). For the immobilized enzyme method, the end repair step was either performed for 30 min at 20°C or 37°C, followed by separation of the enzymes conjugated onto magnetic beads and the supernatant. 3′ A-tailing was carried out by incubation of the supernatant with immobilized Taq DNA polymerase at 37°C for 30 min in dAQ buffer (supplemented with PEG). Library construction using the commercial library preparation kits followed the protocol recommended by the manufacturers. The libraries were analyzed to determine the size distribution using an Agilent High Sensitivity DNA Kit on a Bioanalyzer 2100. The library yield was determined by qPCR using the NEBNext Library Quant Kit for Illumina.

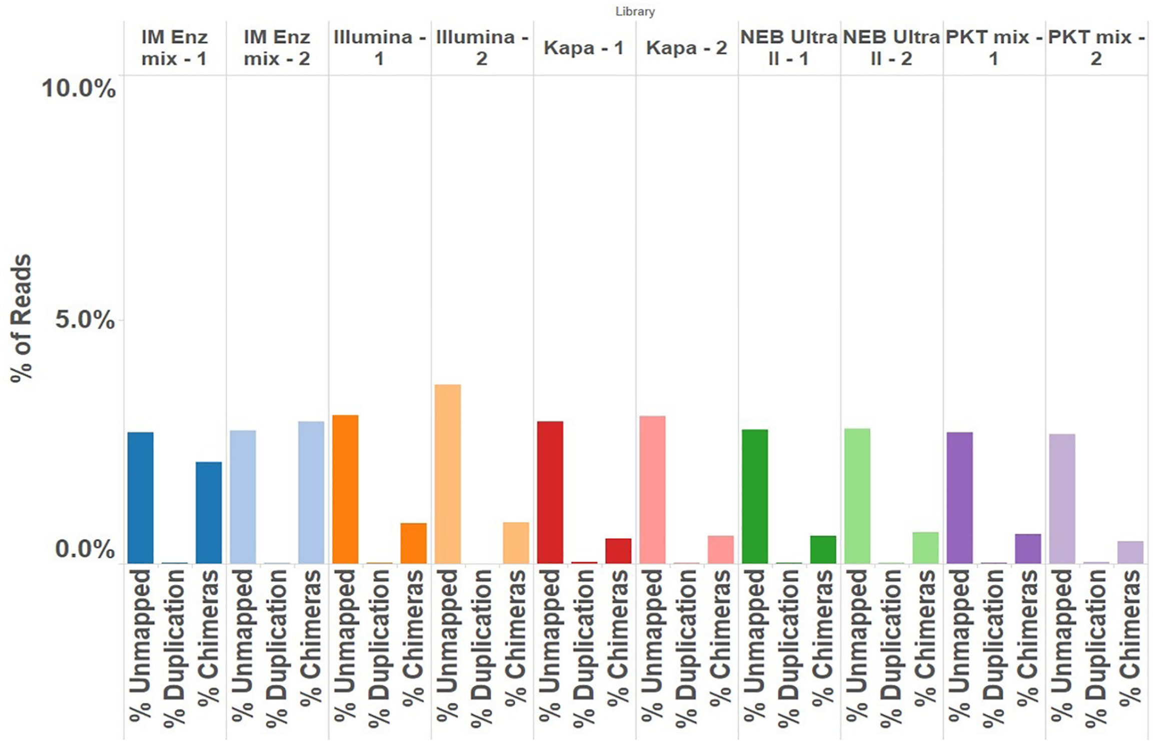

Supplementary Figure 10. Comparison of various metrics of Illumina MiSeq sequencing quality. The percentages of unmapped reads, duplication and chimeras from the libraries prepared by various methods are compared. The immobilized enzymes method yields a higher percentage of chimeric reads compared to Illumina TruSeq DNA PCR-free LT Library Preparation Kit (Illumina), Kapa Hyper Prep Kit (Kapa), NEBNext Ultra II Kit (NEB Ulta II) or the PKT mix; this may be caused by lower 3′ A-tailing activity resulting in higher blunt-end ligation of DNA fragments in the libraries.

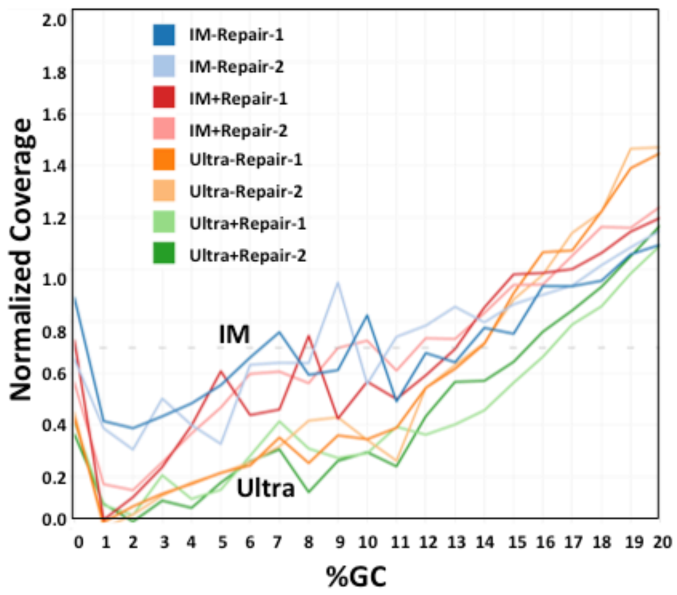

Supplementary Figure 11. Comparison of GC-bias curves of FFPE DNA libraries. Duplicate libraries of FFPE kidney tumor genomic DNA were prepared using immobilized enzyme method (IM) or NEBNext Ultra II DNA library workflow (Ultra) with (+Repair) or without (-Repair) treatment with NEBNext FFPE DNA Repair Mix. These FFPE libraries were amplified by 8 cycles of PCR and paired-end sequenced on an Illumina MiSeq platform. Shown are the normalized sequence coverage curves in AT-rich regions (<20%GC).

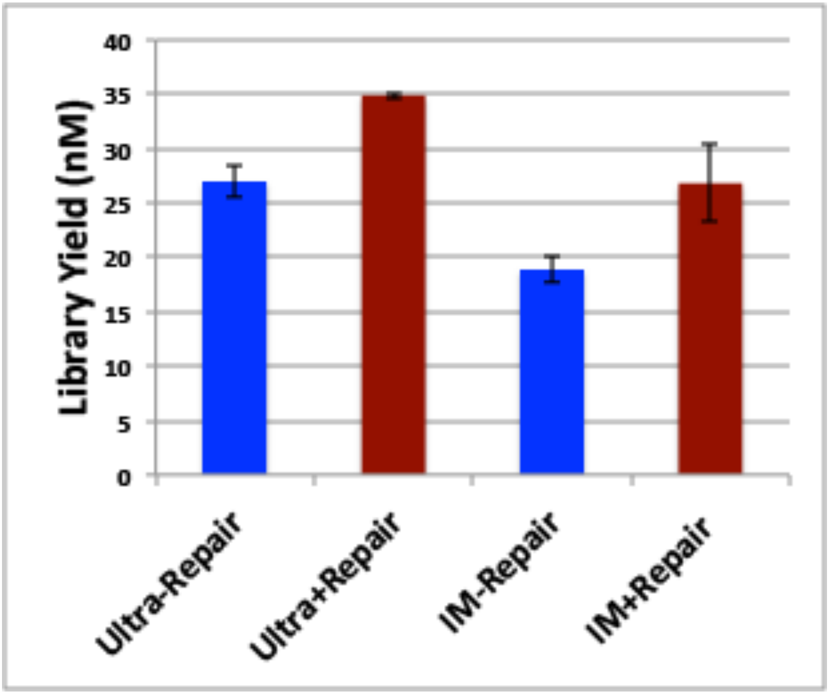

Supplementary Figure 12. Comparison of yields of FFPE DNA libraries. Libraries were prepared by soluble enzymes or immobilized enzymes with or without repair using NEBNext FFPE DNA Repair Mix[17]. Library construction using soluble enzyme mixture (NEBNext Ultra II) for DNA end repair and A-tailing increased library yield by 30% with DNA repair (Ultra+Repair) compared to the protocol with no repair (Ultra-Repair). Use of immobilized enzymes (IM) yielded a 42% increase in library yield with DNA repair (IM+Repair) compared to no repair (IM-Repair).

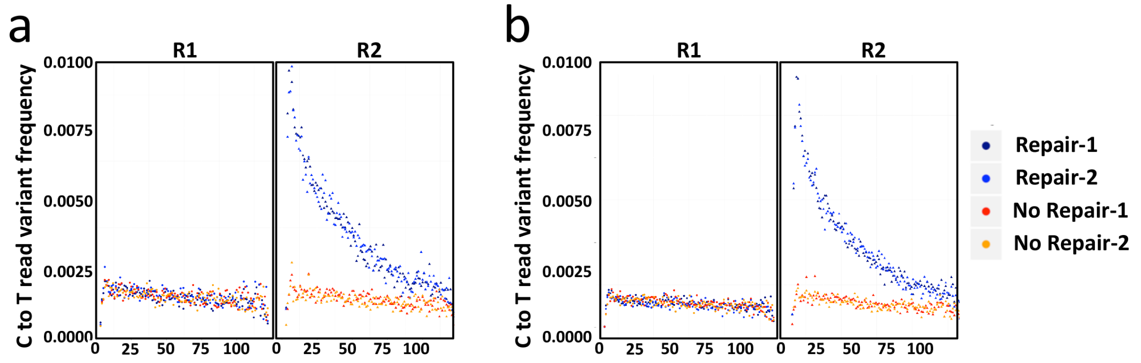

Supplementary Figure 13. C to T read variant rates in repaired and non-repaired FFPE samples. Rate of C to T variants in sequencing reads function of the position on the reads (in bp) in read 1 (R1) and read 2 (R2) in paired-end sequencing of libraries prepared using immobilized enzymes (a) or soluble enzymes (NEBNext Ultra II) (b).
